# Study on dynamic response characteristics of impact of freeze-thaw saturated marble in plateau area

**DOI:** 10.1371/journal.pone.0290628

**Published:** 2023-09-08

**Authors:** Wuhu Huang, Jiandong Yin, Xianglong Li, Defeng Hou, Jianguo Wang, Zhiping Zhang, Ting Zuo, Ximing Jian, Wendong Li, Yang Yang

**Affiliations:** 1 Faculty of Land Resources Engineering, Kunming University of Science and Technology, Kunming, Yunnan, China; 2 Serbia Zijin Copper Doo Bor, Bor, Serbia; 3 Shougang Group Limited Mining Company, Tangshan, Hebei, China; 4 School of Civil and Resource Engineering, University of Science and Technology Beijing, Beijing, China; University of Sharjah, UNITED ARAB EMIRATES

## Abstract

To study the effects that the perennial freeze–thaw environment exerts on the dynamic mechanical properties of marble, which characterizes the Qinghai-Tibet Plateau, impact tests were conducted, and saturated marble was utilized; thus, we analyzed the effect of different loading rates on its dynamic compressive strength, fragmentation pattern, and energy-absorbing density. The results indicate the following: (1) When 42.02s^-1^ ≤$\dot{\varepsilon }$≤ 49.20s^-1^, the degree of fragmentation and the fractal dimension of saturated state marble is greater than that of the dry state marble; when $\dot{\varepsilon }$<42.02s^-1^ or $\dot{\varepsilon }$>49.20s-^1^, the dry state marble exhibits greater fragmentation than the saturated marble; (2) When the saturated state marble is subjected to a specific fractal dimension, the energy-absorbing density of the marble that characterizes the saturated state is great compared with the dry state, and when the fractal dimension increases, the energy-absorbing densities that characterize the two states gradually converge. (3) The effect of water on the mechanical properties of marble has an obvious rate dependence, showing a weakening effect at low strain rates and a strengthening effect at high strain rates. In regard to the analysis pertaining to the dynamic fracture mechanism of marble under the influence of the freeze-thaw environment that characterizes the plateau, the aforementioned experimental results exhibit considerable significance.

## 1. Introduction

Due to years of freeze–thaw cycles, the dynamic mechanical characteristics of the rocks that characterize alpine environments have changed considerably. Furthermore, throughout the mining process, groundwater is frequently encountered, and an immense amount of rock becomes saturated with water. Because they are a non-homogeneous material, rocks exhibit numerous small fractures, which permeate their internal structure. Therefore, it is crucial to analyze the dynamic mechanical properties of the rocks that characterize a saturated state, which pervade mining plateaus, earthquake prediction, tunnel and rock explosion [1–5].

Numerous local and international scholars have immensely analyzed saturated rocks; thus, the physical and mechanical properties of saturated rocks have been somewhat revealed. A.M. Rubin [6] analyzed the dynamic tensile damage that affects the limestone that is subjected to saturated and dry states, and the researcher utilized plane impact tests, which indicated that the dynamic fracture toughness of limestone is robust against both the saturated and dry states. Using laboratory mechanical tests, B. Vásárhelyi [7] studied the mechanical properties of the dry limestone and that of the saturated limestone, and the researcher observed that the effect of saturation on the physical parameters of different rocks remains constant. Using impact tests, Eunhye [8] analyzed the effect of loading rate and water content on the physical and mechanical properties of sandstone, and the researcher observed that the peak stress of the dry sandstone and that of the saturated sandstone was immensely correlated with porosity and bulk density, and that the effect of bulk density on dry sandstone was greater than that of saturated sandstone. GUO [9] tested the tensile strength of sandstone specimens under different dry–wet cycles, and they noted that the tensile strength was immensely correlated the number of dry–wet cycles and the content of clay minerals that characterize the samples. Petr V [10] Using an ultrasonic detection method that utilized shear polarization waves, Petr V tested three coal samples; the researcher analyzed the spectrum of the received signals; thus, it was confirmed that in regard to coal, cyclic freeze–thaw can contribute to the formation and development of cracks. Mousavi [11–13] analyzed rocks that were subjected to freeze–thaw cycles, and the researcher observed that when the number of freeze–thaw cycles increased, the compressive strength and elastic modulus of the rocks decreased, and the following empirical equations, which can be applied to different freeze–thaw cycles were proposed. In summary, foreign scholars have analyzed the mechanical and energy dissipation properties of the rocks that are subjected to dynamic loading, and they have considered the strength, peak stress, and energy dissipation; thus, the energy dissipation law and the damage mechanism that occurs when rocks are damaged by water have been revealed.

To reveal the microscopic mechanisms and critical phenomena that characterize the softening of saturated soft rocks, Cuiying Zhou [14, 15] utilized two numerical analysis methods, namely nonlinear dynamics and scanning electron microscopy. Ma [16] analyzed the bulkiness distribution and fractal dimension of stabilized soil that is subjected to impact conditions, and the researcher observed that the bulkiness distribution of stabilized soil satisfied the fractal law, and that when the sand content in-creased, the fractal dimension increased. Lv [17] observed that in regard to calcareous sand, the degree of crushing was positively correlated with impact energy and negatively correlated with water content, and in regard to calcareous sand, the researcher determined the parameters that determine the degree of crushing. Bin Wang [18] per-formed impact tests on sandstones, and the researcher considered dry and saturated conditions; thus, the researcher concluded that the degree of damage to the saturated sandstones under dynamic loading conditions is considerably lower than that to sand-stones under dry air-dry conditions. Feng Fan [19] performed a true triaxial dynamic disturbance test, and the researcher concluded that the sandstone, in its dry state, mainly exhibits mixed tension–shear damage, and that the saturated sandstone exhibits tension damage. Yunsi Liu [20] performed impact tests on satiated slate, and the researcher observed that the energy dissipation density of dry slate is considerably lower than that of satiated slate. Hengyuan Zhang [21] performed a series of experiments including the uniaxial compression of granite in different water-bearing states; thus, the researcher revealed the fracture evolution mechanism and crack expansion law of water-bearing rocks, and it was observed that water exerts a significant weakening effect on the strength and elastic modulus of the rocks. In summary, using microscopic mechanisms and impact tests, domestic scholars have explored the effect of water on the physical and mechanical properties of rocks, and by analyzing the damage characteristics and fractal properties of rocks, they have obtained practically significant conclusions. By studying the effect of water on the physical properties of rocks, Zhang [22] concluded that the modulus of elasticity and strength of rocks decrease as the water content increases.

The aforementioned study indicates that with respect to rocks, water affects the physical and mechanical properties, energy dissipation properties, and fractal fracture. Herein, uniaxial impact tests, which utilize different loading rates, were conducted on dry and saturated marble; the marble was obtained from the Niu Kutou mining area that is situated in the Qinghai plateau region, and using a split Hopkinson com-pression bar, the energy transfer law of marble under different strain rates was explored, the fractal law of marble mass after impact crushing was analyzed, the relationship between the energy dissipation that characterizes marble crushing under impact load-ing and fractal dimension was constructed, and the dynamic strength characteristics of marble in different regions was compared; thus, a theoretical guidance for comprehending the dynamic mechanical properties of saturated rocks, which characterize the plateau freeze-thaw environment, was developed.

## 2. Test preparation and program

### 2.1. Specimen preparation

In regard to this test, the marble that was utilized was obtained from the Niu Kutou mining area; the mining area is located on the north slope of Qimantag Mountain, which is located in the northeastern part of the Qinghai-Tibet Plateau, a typical alpine area with an average annual temperature of 4.3°C. The groundwater veins are somewhat developed, and they belong to the saturated section. To ensure that the non-parallelism and non-perpendicularity of the specimen ends were less than 0.02 mm, the specimen ends were sanded and polished [23, 24].

**Fig 1:**
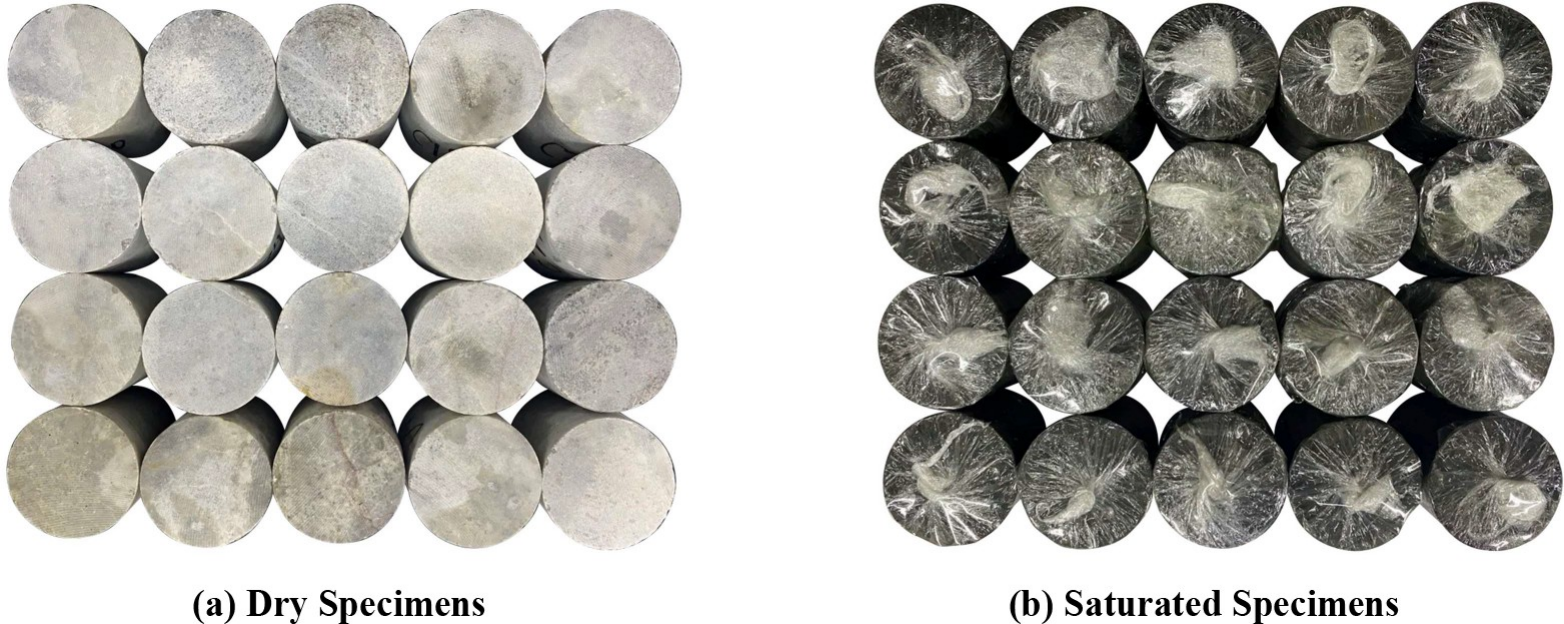
Marble specimens.

The basic mechanical parameters of the specimen are depicted in Table 1, and Fig 1 indicates that the specimen is moulded into a cylindrical shape with a 50 mm diameter and a 50 mm height (length-to-diameter ratio; 1).

**Table 1:** Basic static parameters of marble.

State	Angle of Density/g∙cm^-3^	Compressive strength/MPa	P-wave Velocity (m/s)	Poisson’s ratio	Modulus of elasticity/GPa
Dry	2.70	50.24	6082	0.24	42.65
Saturated	2.69	49.12	6154	0.26	---

### 2.2. Test program

**Fig 2:**
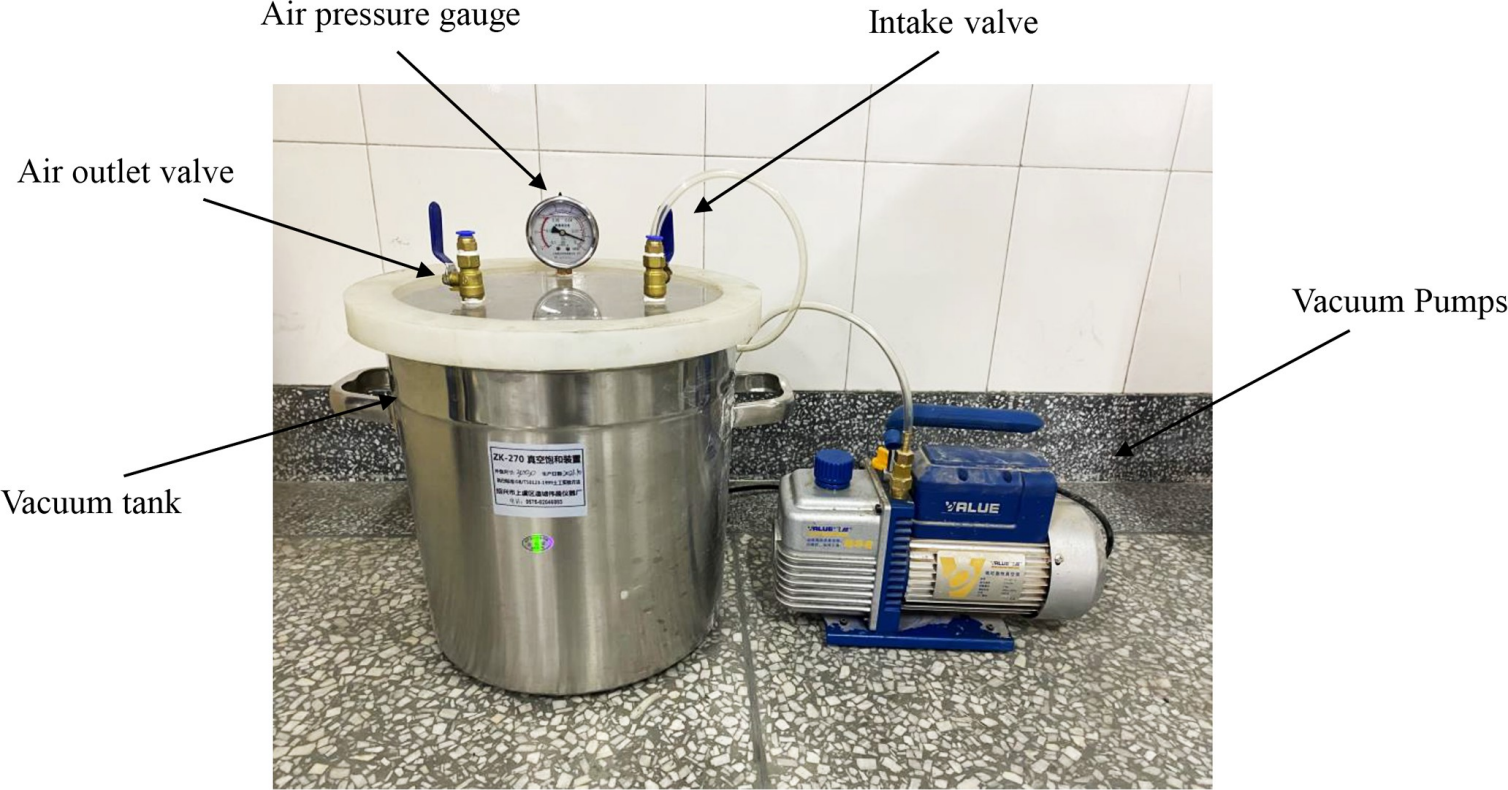
Full water test equipment.

In regard to this test, the kinetic SHPB test system that was recommended by the International Society of Rock Mechanics was utilized, and in regard to the determination of rock kinetic parameters, this equipment has been widely utilized [22, 23]. Fig 2 depicts the full water test equipment. Using the vacuum-forced full of water method, test the full water specimen; insert the prepared specimen into the vacuum tank, and add water to submerge the specimen; to pump out the air that is inside the vacuum tank, run the machine; the number of hours as a whole gradient, each full hour, the test piece out of weighing once, until the quality of the test piece no longer changes, which indicates that the test piece has attained the full water state. In regard to this test, the pump was operated for a total of 6 hours, and the specimens attained a full water state. To ensure that the final effect of the test is exerted, a test punch test is required; thus, the range of impact air pressure can be determined. It was observed that when the air pressure was 0.25 MPa, the specimen was gradually damaged; therefore, the impact test was performed; furthermore, the impact air pressure ranged from 0.25 MPa to 0.45 MPa, and the increment pertaining to the impact air pressure was 0.05 MPa. The specimen is coated with grease at the contact end of the incident rod and that of the transmission rod; thus, the effect that friction exerts on the test effect is reduced and the test accuracy is enhanced [24].

## 3. Test analysis of the test results

### 3.1. Determination of stress equilibrium and strain rate

The initial air pressure was set at 0.25 MPa, and by gradually increasing the gradient by 0.05 MPa, the impact test was performed on saturated marble and dry marble.

**Fig 3:**
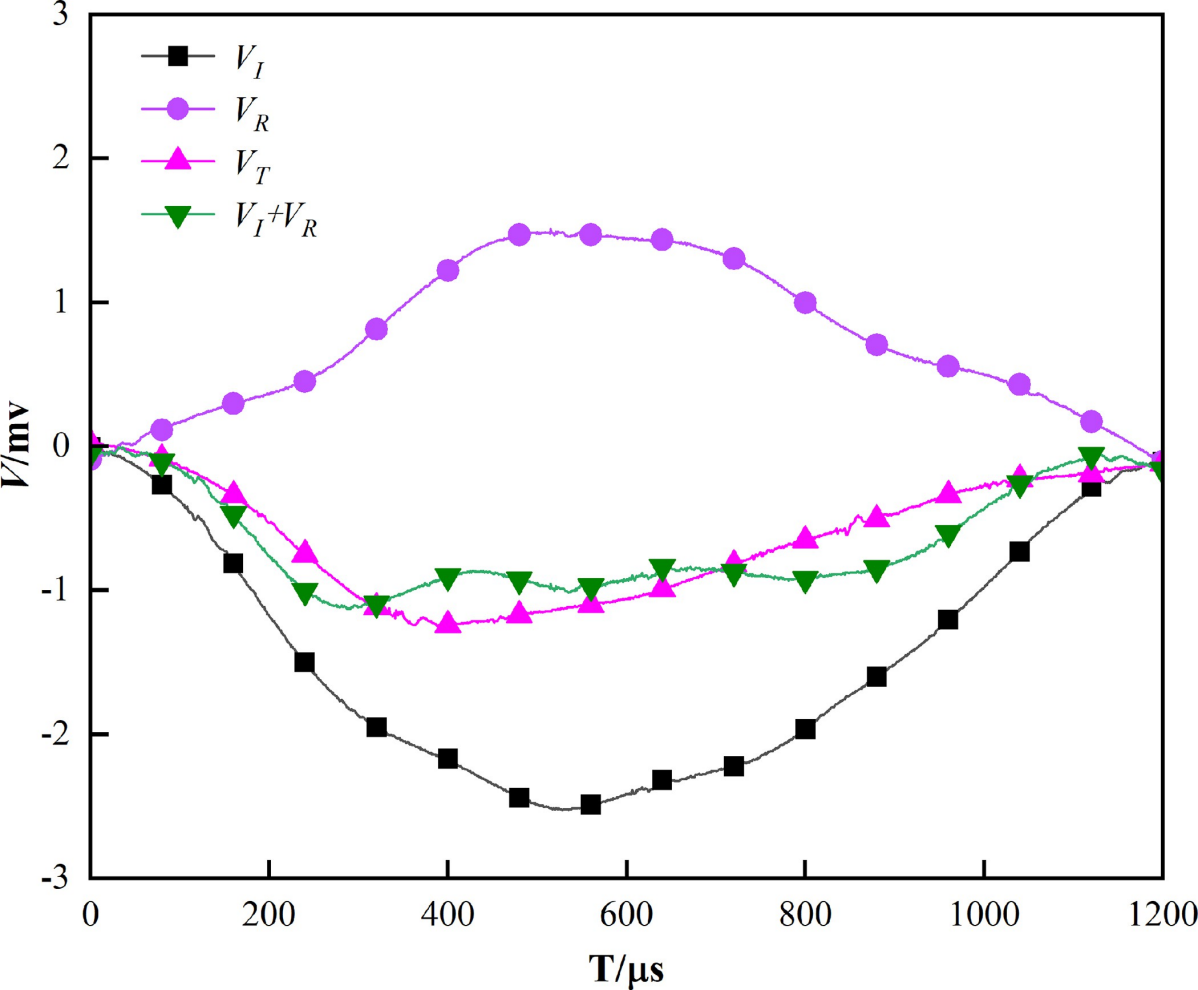
Voltage balance diagram.

Based on the one-dimensional stress propagation theory, we can determine the rationality of the SHPB test, and to ensure the reliability of the test, the stress balance principle must be followed [25, 26]. Therefore, by comparing the transmitted voltage (*V*_*T*_) with the incident voltage and reflected voltage (*V*_*I*_*+V*_*R*_) in the original waveform, dis-carding the unqualified data, and making up the corresponding test, this study examines the stress wave balance. Fig 3 illustrates the voltage balance diagram that was obtained by shifting and summing the incident, transmitted, and reflected voltages. By observing the Fig 3, we can note that the novel *V*_*I*_*+V*_*R*_ waveform approximates the *V*_*T*_ waveform, which indicate the test voltage balance.

Three sets of impact tests were conducted at each air pressure, and for analysis, the data that was obtained from the three tests was averaged. Using the three-wave method, the stress–strain rates were calculated [27–29]. Using the one-dimensional stress wave assumption, the incident, transmitted, and reflected energies that were emitted during the whole test were obtained [30, 31]. The energy dissipation that occurs between the rock specimen and the compression bar is not considered during the test, and the energy absorption density can be calculated as per the energy conservation law [32, 33]. The calculation results are show in Table 2.

**Table 2:** Rock strength and energy parameters under different loading conditions.

Rock state	Loading parameters	Strength parameters			Energy Parameters		
	Strain rate/(s^-1^)	Dynamic compressive strength /MPa	Failure strain	DIF	Dissipated energy /(J)	Energy absorption rate	Energy absorption density /(J·cm^3^)
Dry	28.32	85.88	0.0041	1.71	24.51	0.27	0.25
	39.71	94.44	0.0013	1.88	32.54	0.25	0.39
	44.94	99.95	0.0088	1.99	42.2	0.26	0.52
	51.45	109.73	0.0034	2.18	53.27	0.29	0.64
	57.48	126.73	0.0025	2.52	78.37	0.36	0.95
Saturated	29.33	59.69	0.0026	1.22	19.37	0.24	0.26
	36.65	73.72	0.0039	1.50	31.49	0.25	0.42
	46.72	82.78	0.0023	1.68	41.85	0.27	0.55
	52.22	88.98	0.0045	1.81	50.05	0.27	0.69
	59.32	115.42	0.0026	2.35	76.8	0.36	1.00

**Fig 4:**
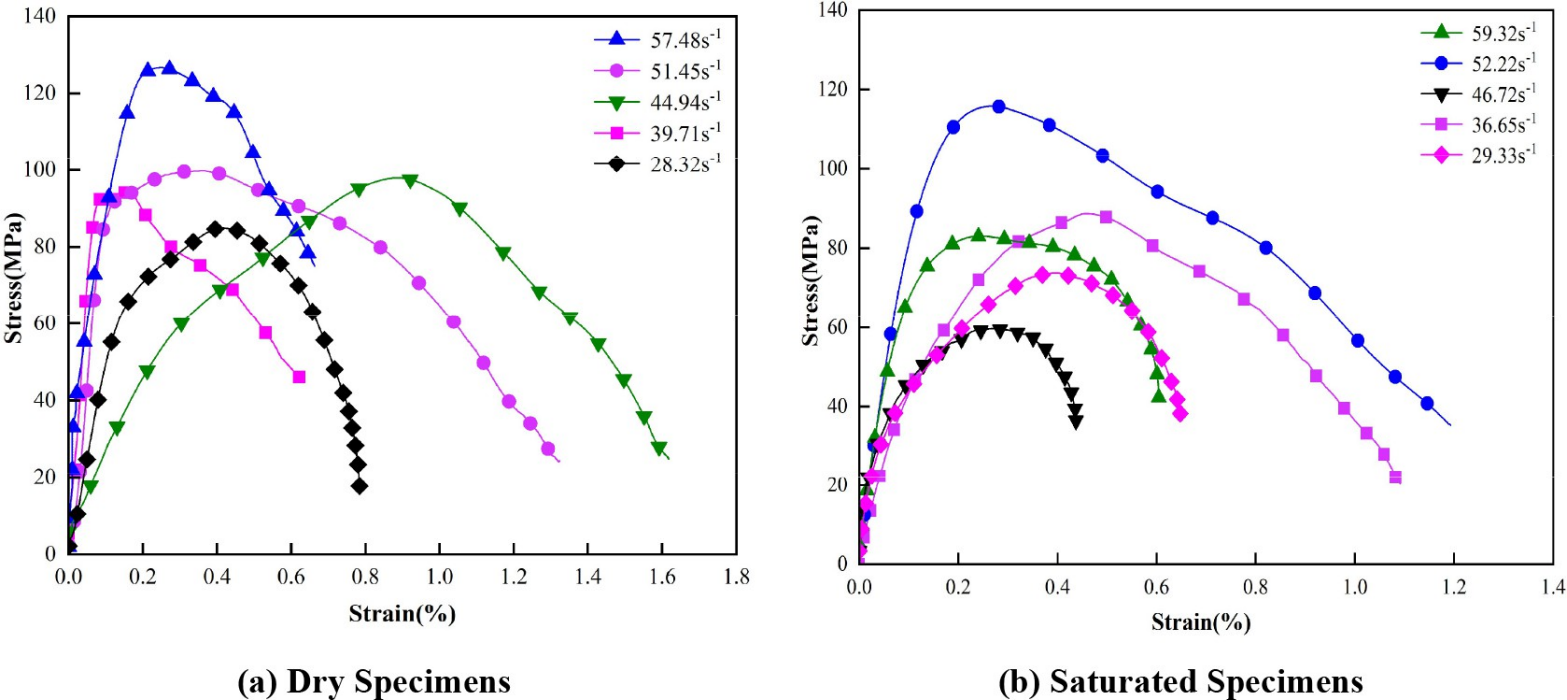
Stress–strain curves pertaining to marble that is subjected to different strain rates.

The stress–strain curves pertaining to the uniaxial impact tests that were conducted on dry marble and saturated marble are depicted in Fig 4.

**Fig 5:**
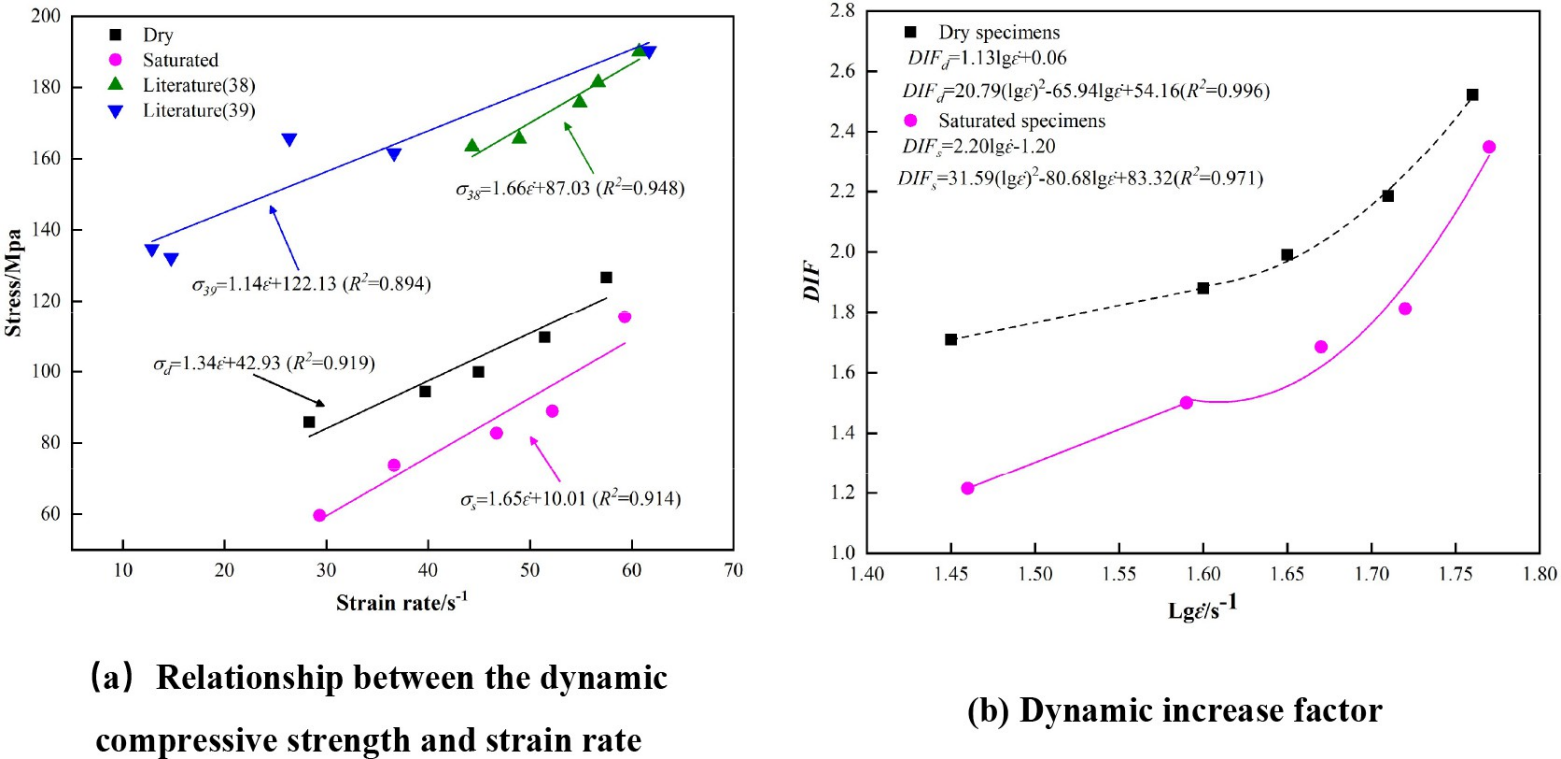
Dynamic compressive strength and dynamic increase factor with respect to strain rate.

Fig 4 indicates that the pattern pertaining to the stress–strain curves of the marble specimens that are subjected to different states and different loading strain rates remains constant. In regard to the initial stage, the stress–strain curves of the marble are roughly linear; furthermore, the deviations are not large, and the two curves can effectively coincide, which indicates that in regard to this stage, the rocks pertaining to the two states exhibit satisfactory linear elasticity characteristics. When the strain rate increases, the peak stress value of the stress–strain curve also increases. When the rock samples are subjected to similar strain rates, the peak stresses that characterize the saturated specimens are generally low compared to those that characterize the dry state, which indicates that water reduces rock strength. When the rocks specimens are subjected to impact loading, a large number of nascent cracks will also facilitate the damage deformation of the rock, which can lead to an increase in the macroscopic rupture surface of the rock specimen, which occurs after rock damage; a decrease in the bearing capacity of the rock; a significant softening effect; and, finally, a decrease in the peak strength of the rock specimen. Numerous experimental studies have confirmed that the dynamic compressive strength of rocks apparently exhibits a positive correlation with the loading rate or strain rate in a certain range, and that when the strain rate increases, the dynamic compressive strength of the rocks increases [34, 35]. The fitted relationship between the dynamic compressive strength and the average strain rate pertaining to the rock specimens that are subjected to different states is depicted in Fig 5(A) [36, 37].

### 3.2. Effect of strain rate and water on compressive strength

As depicted in Fig 5(A), the specimen that is utilized in Literature 38 is dolomite marble (dry state) from the roadway envelope that is located in Dahongshan, Yunnan Province, and the specimen that is utilized in Literature 39 is marble (dry state), which is obtained from Jinping Hydropower Station, Guizhou Province [38, 39]. The compressive strength of both the dolomite marble and the marble that is obtained from Jinping Hydropower Station considerably exceed that of the marble from the Niu Kutou mine; this phenomenon may be occasioned by the large diurnal temperature difference that characterizes the alpine region and the formation of the freeze–thaw effect on rock strength, which is highly significant. When the ambient temperature is low, the freezing effect of pore water and the formation of freezing and swelling forces expand the rock volume, which leads to local damage inside the rock. When the ambient temperature is high, the water inside the rock gradually dissolves, and the freezing expansion force is released. After several freeze–thaw cycles, the strength of the rock is continuously reduced [40–42].

It is indicated that the dynamic compressive strength of the rock specimens that are subjected to both states exhibits a satisfactory linear relationship with the average strain rate. After water saturation, the strength of the rocks weakened significantly, and after water saturation treatment, the dynamic compressive strength of the marble was reduced by approximately 10.42%~22.61%, which may be occasioned by the following: after the water-filled test, the water reduces the cementation between the mineral particles that constitute the rock, and when the sample is subjected to external loading, the free water that is contained inside the specimen travels to the crack tip and forms a “water wedge”, which accelerates the crack expansion and damages the specimen. Several of these factors may have contributed to the following observation: the dynamic compressive strength of saturated marble is considerably lower than that of the dry state.

The dynamic increase factor (*DIF*), the ratio of dynamic peak stress to static peak stress, is introduced to quantify the rate dependence of marble strength [24]. The *DIF* values in Table 2 can be determined by the compressive strengths of dry and saturated marble in Table 1, and the *DIF* values diagram at different strain rates are created, as illustrated in Fig 5(B).

The *DIF* value of dry marble increases by 1.01 when the strain rate is between 28.32s^-1^ and 57.48s^-1^; The *DIF* value of saturated marble increases by 1.07 when the strain rate ranges from 29.33s^-1^ to 59.32s^-1^, and saturated marble exhibits a more pronounced rate correlation. Linear and quadratic functions (formulas (1) and (2), respectively) can be used to characterize the dynamic strength factor variation trends of dry and saturated marble.


$$\left\{ \begin{array}{l} DIF_{d}=1.31\mathrm{lg}\dot{\varepsilon }+0.06(28.32s^{-1}\leq \dot{\varepsilon }<39.71s^{-1})\\ DIF_{d}=20.79{\left(\mathrm{lg}\dot{\varepsilon }\right)}^{2}-65.94\mathrm{lg}\dot{\varepsilon }+54.16(39.71s^{-1}\leq \dot{\varepsilon }\leq 57.48s^{-1}) \end{array}\right.$$ (1)

$$\left\{ \begin{array}{l} DIF_{s}=2.20\mathrm{lg}\dot{\varepsilon }-1.20(29.33s^{-1}\leq \dot{\varepsilon }<36.65s^{-1})\\ DIF_{s}=31.59{\left(\mathrm{lg}\dot{\varepsilon }\right)}^{2}-80.68\mathrm{lg}\dot{\varepsilon }+83.32(36.65s^{-1}\leq \dot{\varepsilon }<59.32s^{-1}) \end{array}\right.$$ (2)

**Fig 6:**
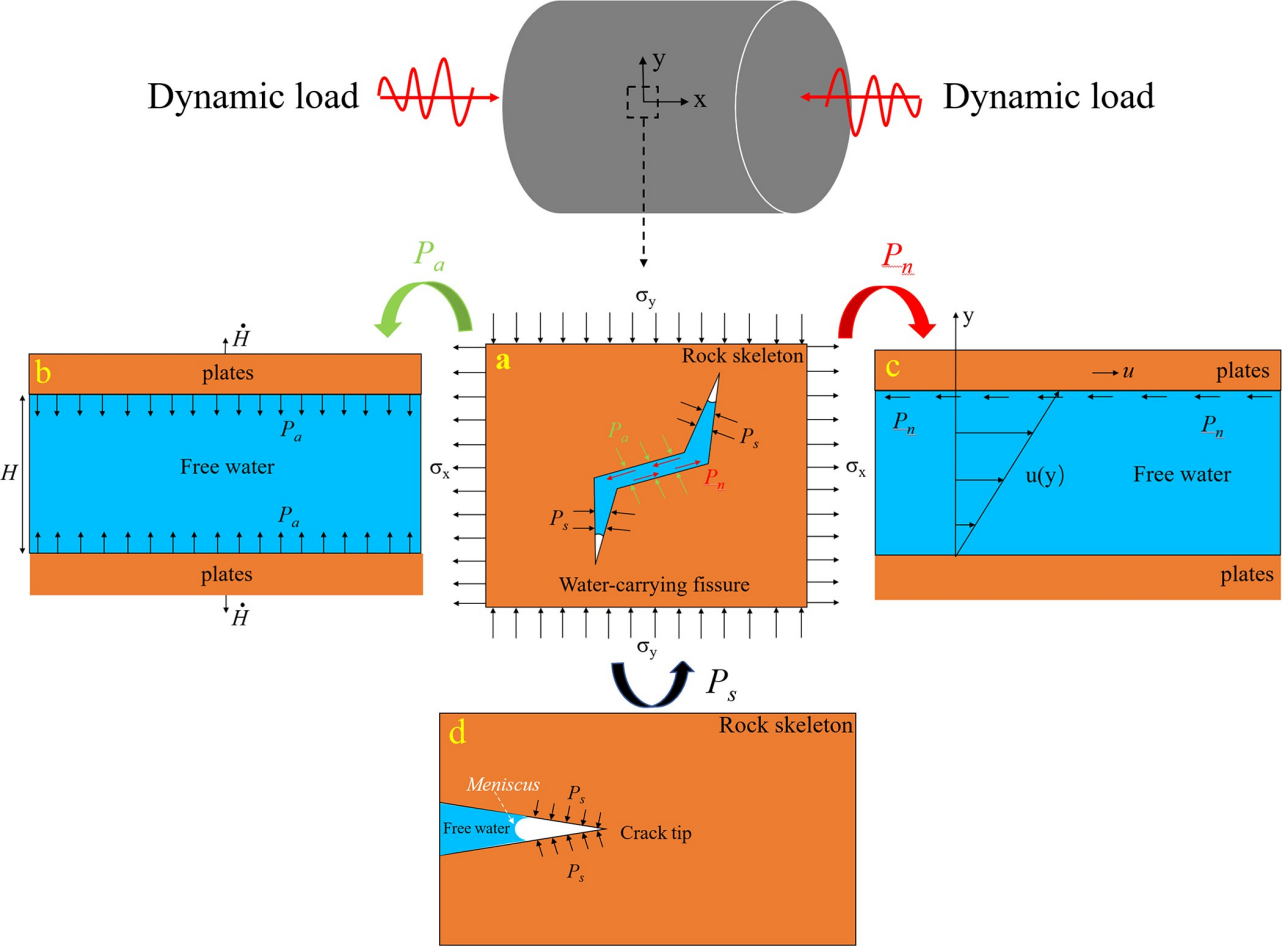
Water effect on rock under coupled dynamic loading. (a) Extra resisting force induced by free water under dynamic loading; (b) Stefan effect; (c) Schematic of newton inner friction effect; (d) Meniscus effect.

According to the relevant research, water plays a weakening role in the strength of rock when the strain rate is within a certain range [44, 45]. The above results show that the dynamic compressive strength of saturated marble has a strong correlation with strain rate, which is the result of the combined action of the Stefan effect, the schematic of the Newton inner friction effect, and the Meniscus effect under the influence of strain rate. As shown in Fig 6 below, the resisting stresses (*Pn*, *Ps*, and *Pa*) formed by the above three effects act on the main crack and wing crack, respectively: (1) *Pn*, as shown in Fig 6(B), when the loading rate is higher, the resistance caused by the Stefan effect is greater, which inhibits the initiation and expansion of cracks [43, 44].(2) *Ps*, as shown in Fig 6(C), when the relative sliding velocity of the crack surface is greater, the resisting stress caused by the Schematic of newton inner friction effect is greater, resulting in enhanced friction strength [45]. (3) *Pa*, as shown in Fig 6(D), the free water will form a meniscus at the top of the wing-shaped crack and generate a pulling force on the crack to inhibit the crack propagation [46].

### 3.3. Effect of strain rate and water on energy dissipation

**Fig 7:**
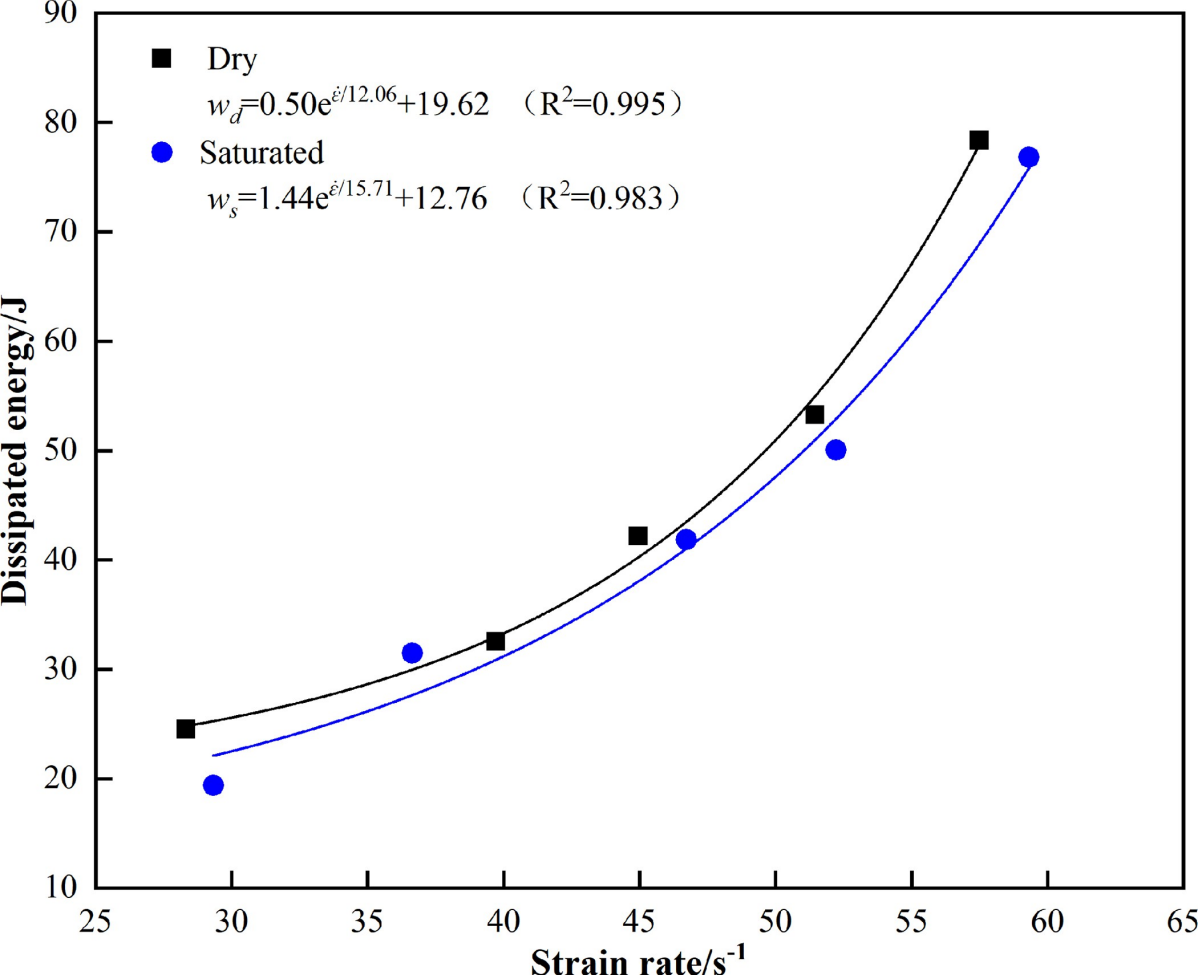
Relationship between dissipated energy and strain rate in different states.

Fig 7 is a graph showing the relationship between the dissipated energy of marble and water under different strain rates. It can be seen from Fig 7 that the dissipated energy of marble in both states increases with the increase in strain rate, and both of them show a strong rate correlation. The dissipative energy of marble in the dry state is significantly higher than that in the saturated state and shows a stronger rate dependence, which is due to the weakening effect of water that reduces the strength of the marble, lower energy is required for rock failure under external loads.

The energy absorption rate*η*of the marble specimen is the ratio of the dissipated energy *w*_*s*_ of the specimen to the incident energy *w*_*i*_, which characterizes the energy utilization efficiency of the specimen during the dynamic failure process, as shown in the following Formula (3).


$$\eta =\frac{w_{s}}{w_{i}}$$ (3)

**Fig 8:**
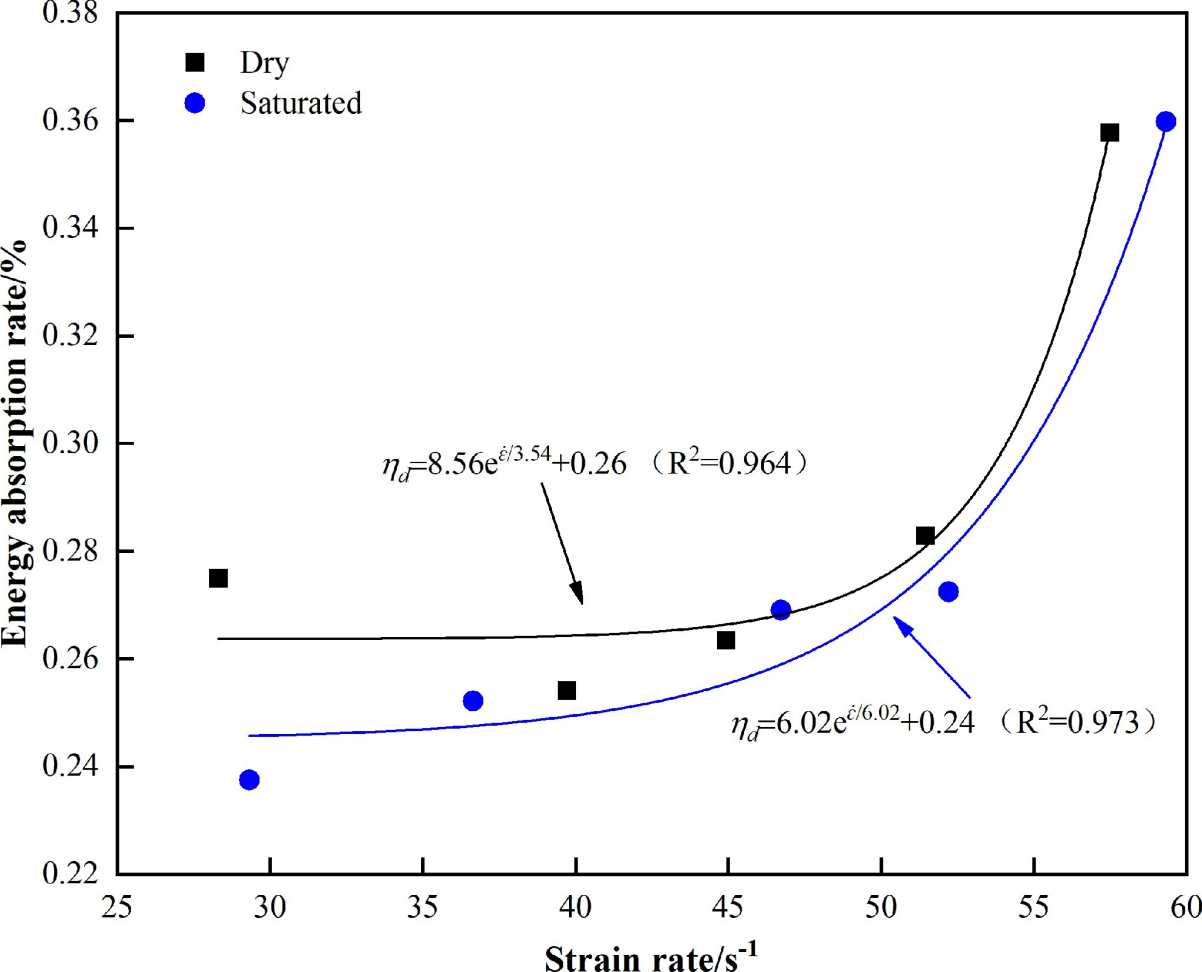
Relationship between energy absorption rate and strain rate in different states.

In order to study the relationship between the energy absorption rate and strain rate of the specimens under different states, Fig 8 is drawn. It can be seen from the Fig 8 below that the energy absorption rate of the specimens in both states shows an upward trend with the increase of the strain rate, and the energy absorption rate of the dry state is higher than that in the saturated state as a whole. This means that water will reduce the energy absorption rate of rocks. At this time, the weakening effect of water is dominant. With the increase in the strain rate, the difference of the energy absorption rate between the two states decreases gradually, indicating that the weakening effect of water is weakening. When the strain rate exceeds a certain range, the difference between the two will gradually increase. From the perspective of energy absorption, it can be seen that the strain rate has a certain influence on the mechanism of water.

### 3.4. Damage characterization

**Fig 9:**
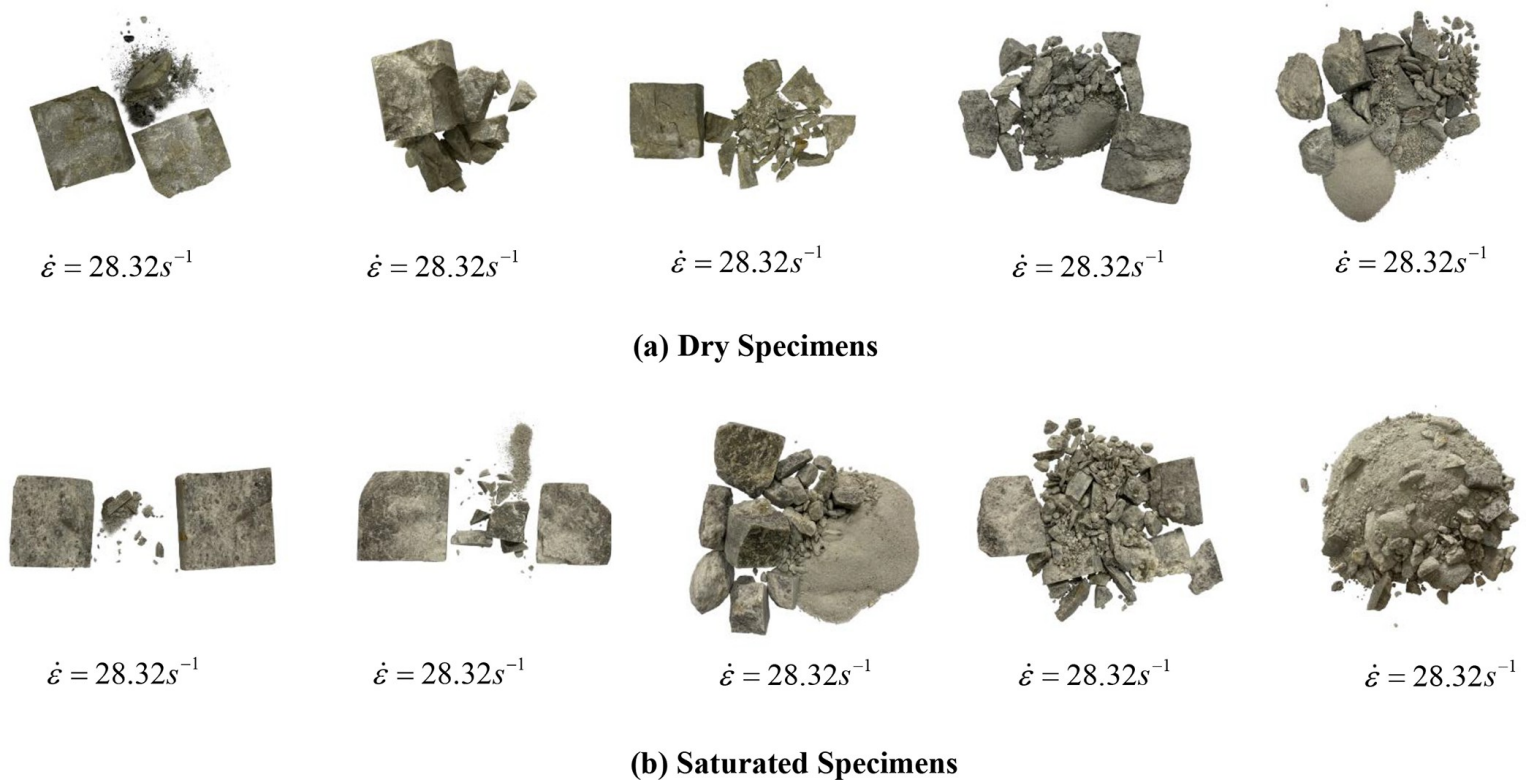
Damage pattern of marble at different impact velocities.

The overall fragmentation characteristics pertaining to the marble that is subjected to the two states at different impact velocities are depicted in Fig 9.

Fig 9(A) and 9(B) illustrate that the marble that is subjected to both states at lower velocities (*v* = 9.40 m/s, *v* = 9.25 m/s) of impact exhibits splitting damage. Due to the in-crease in the average strain rate, the damage morphology exhibits edge crumbling, core retention damage, block damage, and crushing damage. In regard to the fracture morphology, the behaviour of the marble is robust against both states. With respect to the crushed block, when the impact velocity gradually increases, the proportion of large pieces gradually decreases, and the proportion of fine particles gradually increases. The degree of crushing that characterizes the marble in both states also deepens, and the change in the size of the crushed block follows the same pattern. When the energy ab-sorption density increases, the block size decreases, which indicates that the loading rate increases the energy that acts on the rock rupture, and more rupture surfaces are formed inside the rock; thus, the overall block size decreases.

When *v* = 11.58 m/s, the degree of fragmentation that is exhibited by the saturated marble is larger, which may be occasioned by the presence of a soft interlayer, and the soft interlayer that is subjected to dynamic loading is considerably prone to disturbance-based fragmentation; therefore, compared with other specimens, this rock specimen exhibits a large degree of fragmentation.

### 3.5. Rock fractal study

The grading standard sieve is selected, and the pieces are classified into ten kinds of particle-size grades, namely 0~0.3mm, 0.3~0.5mm, 0.5~1.0mm, 1.0~2.5mm, 2.5~5.0mm, 5~10mm, 10~15mm, 15~20mm, 20~25mm, and >25mm. Weigh the pieces that exhibit different particle sizes after screening, the percentage of each particle size is calculated; furthermore, by plotting the distribution curve of rock crushing bulk at different strain rates, the fractal dimension is calculated, as expressed in the following equation [47]:

$$\frac{M(r)}{M(z)}=\frac{M(x<r)}{M(x<r_{z})}={\left(\frac{r}{r_{z}}\right)}^{3-D}$$ (4)

where: *M(r)* denotes the particle mass; *M(z)* denotes the total mass of the specimen; 3-*D* denotes the slope of the lg[*M(r)*/*M(z)*]-lg*r*-fitting curve, set the slope as *b*, where *b* = 3-*D*; *D* denotes the fractal dimension; *r* denotes the characteristic value of the particle size; and *r*_*z*_ denotes the maximum diameter of the fragment. Take the double logarithm on both sides of the above formula to get the following formula.


$$\mathrm{lg}(M(r)/M_{z})=(3‐D)\mathrm{lg}(r/r_{z})$$ (5)

**Fig 10:**
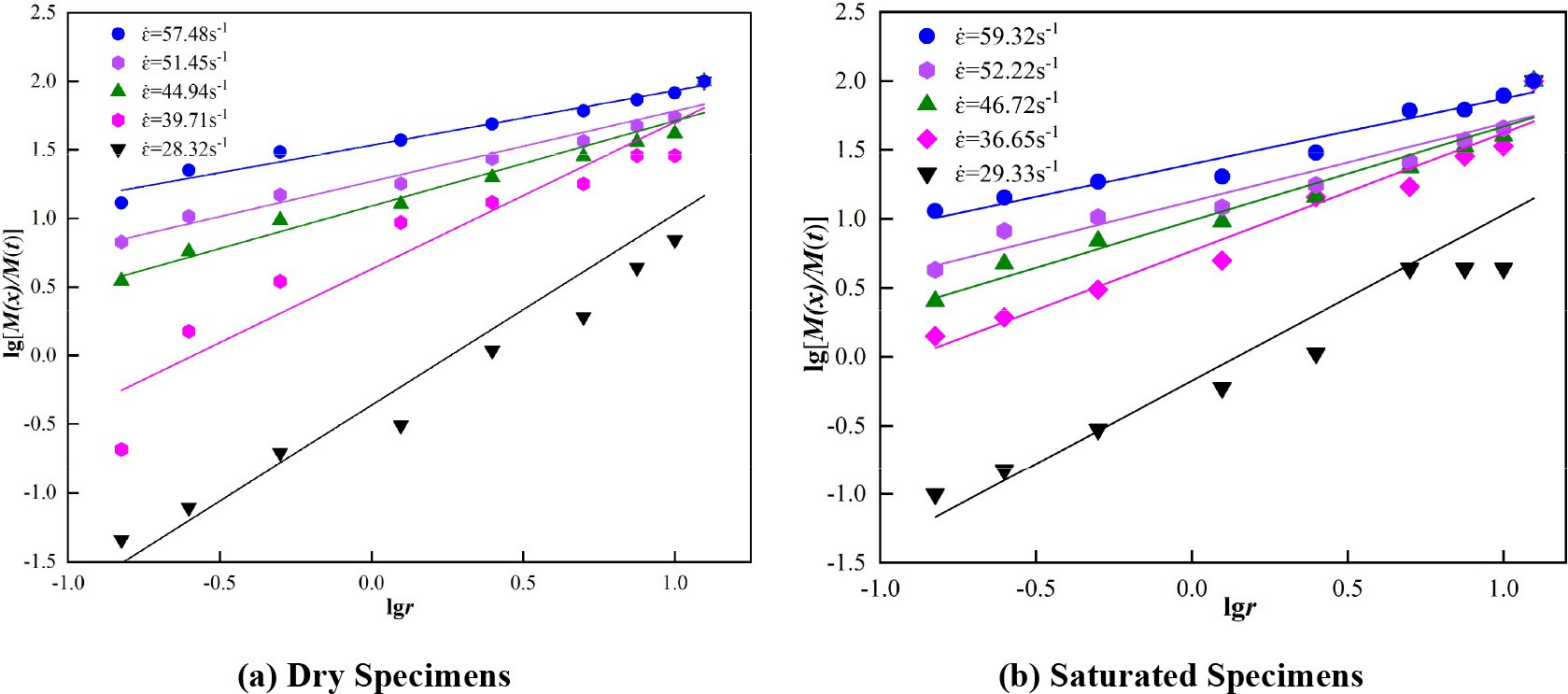
Distribution of marble fracture bulkiness lg[*M(r)*/*M(z)*]-lg*r* curves in different states.

The fragmentation bulk distribution lg[*M(r)*/*M(z)*]-lg*r* curves pertaining to the dry and saturated state marble are depicted in Fig 10.

Fig 10 indicates that in regard to the crushed rock that characterizes the marble pertaining to both states of the double logarithmic coordinate system, the percentage of each particle size and the size of the graded standard sieve exhibited a significantly positive linear correlation. The results of the calculation pertaining to the fractal dimension and correlation coefficient are depicted in Table 3. The correlation coefficients are all large, and the confidence level pertaining to the fitted curves is high, which indicates that the crushing bulk distribution of the marble specimens that were subjected to impact all satisfy the fractal law.

**Table 3:** Calculated values of fractal dimension for different strain rates.

Rock state	Strain rate $\dot{\varepsilon }$/s^-1^	Fractal dimension *D*	Correlation coefficient *R*^*2*^
Natural	28.32	1.80	0.9323
	39.71	2.06	0.9471
	44.94	2.43	0.9708
	51.45	2.53	0.9752
	57.48	2.67	0.9845
Full water	29.33	1.58	0.9090
	36.65	2.04	0.9726
	46.72	2.38	0.9673
	52.22	2.51	0.9518
	59.32	2.59	0.9732

#### 3.5.1. Effect of strain rate on fractal dimension

**Fig 11:**
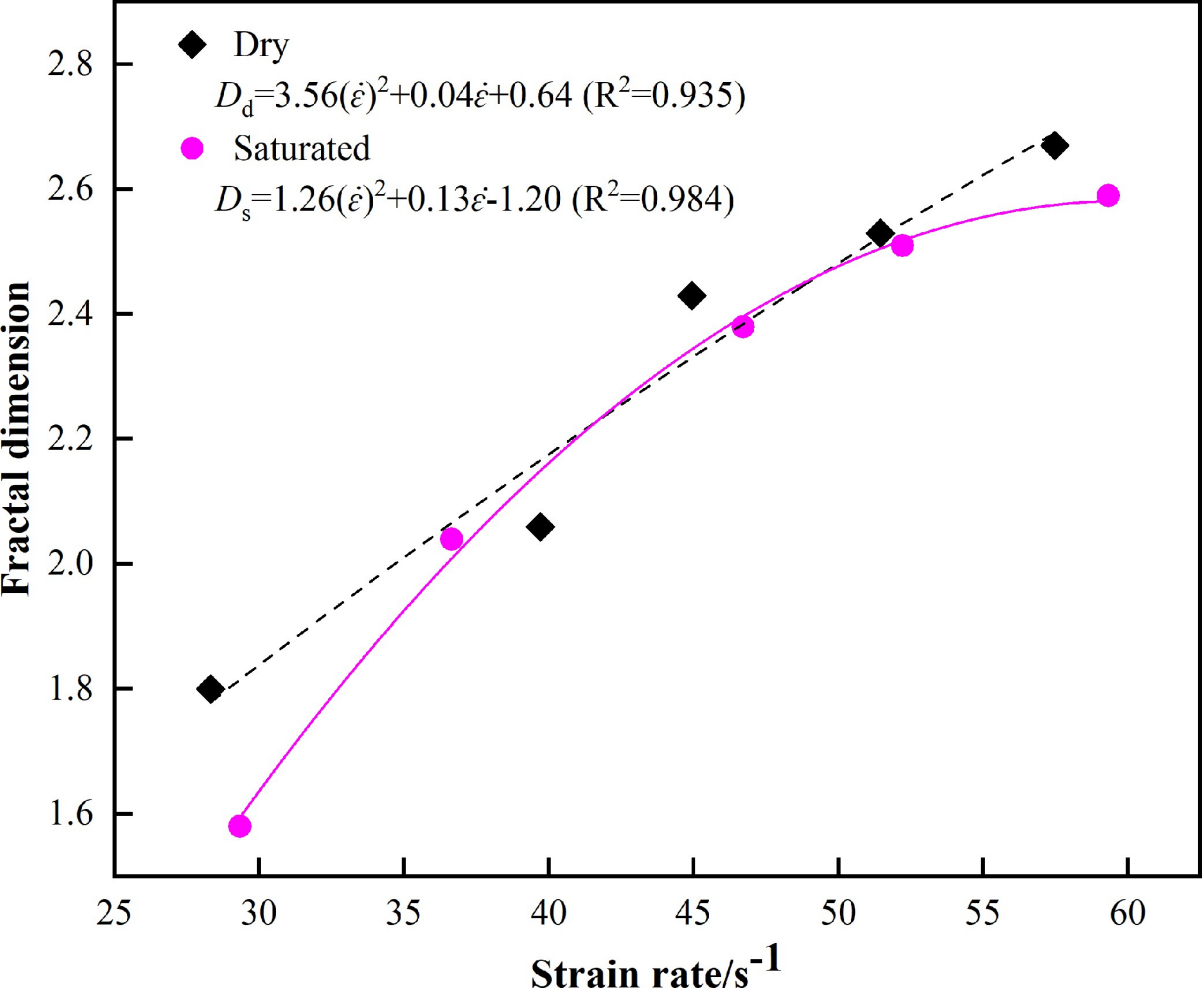
Relationship between the fractal dimension and strain rate.

To study the fractalization and energy dissipation laws of marble under impact loading, draw a scatter diagram of strain rate and fractal dimension and perform fitting analysis on it. The results are depicted in Fig 11.

Fig 11 indicates that when the strain rate increases, the fractal dimension increases, and the two factors exhibit a significant linear correlation. When $\dot{\varepsilon }$≤ 42.02s^-1^, the fractal dimension of the dry state marble is greater than that of the saturated marble; however, the growth rate of the dry state marble is lower than that of the saturated marble, which indicates that during this stage, the inhibitory effect that water exerts on marble fragmentation is weakened; When 42.02s^-1^ ≤ $\dot{\varepsilon }$≤ 49.20s^-1^, the fractal dimension of satiated marble and that of dry marble approximate each other, which indicates that in regard to the aforementioned strain rate range, water exerts a low influence on the fractal fractality of marble; When $\dot{\varepsilon }$>49.20s^-1^, the fractal dimension of dry marble is larger than that of saturated marble, and the difference between the two states becomes larger, which indicates that during this stage, the effect of water on the fragmentation degree of marble is highly significant.

Macroscopically, the degree of fragmentation pertaining to the saturated marble is lower than that of dry marble; under the action of water, the absorbed energy that is transformed by the energy that is transmitted by the stress wave into the interior of the specimen is preferentially distributed to the main cracks inside the rock specimen; the expansion of fine cracks is inhibited, and nascent cracks cannot be effectively produced. The rock becomes damaged, which leads to the dissipation of energy, and this energy fails to comprehensively act on the crack expansion; thus, more large pieces are formed, which leads to the reduction of the fractal dimension value.

#### 3.5.2. Effect of fractal dimension on compressive strength and energy absorption density

**Fig 12:**
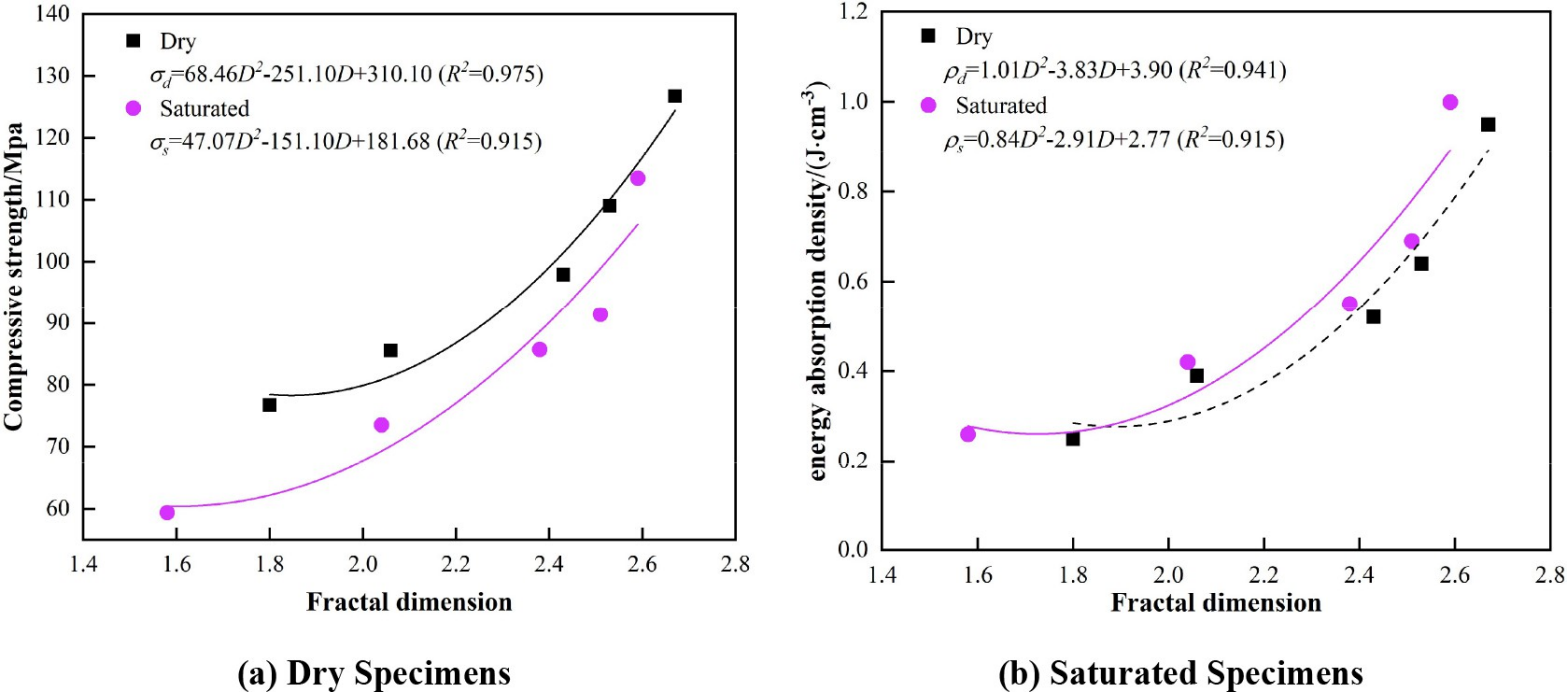
Relationship between fractal dimension and compressive strength and energy absorption density.

To reveal the correlation between the fractal dimension and dynamic compressive strength and the energy-absorbing density pertaining to different states of marble damage, the fractal dimension, compressive strength and energy absorption density of marble are fitted with a quadratic function with good effect and the results are depicted in Fig 12.

Fig 12(A) indicates that when the fractal dimension increases, the dynamic compressive strength of marble increases. If the fitted equations of both marble specimens are compared, it can be observed that with respect to dry state marble, each coefficient is considerably larger than in its saturated state; thus, when the fractal dimensions are constant, the compressive strength of the dry marble is considerably greater than that of the saturated marble, which indicates that water reduces the strength of marble. As the fractal dimension increases, the energy-absorbing density of the marble also increases, and the two exhibit a considerably positive correlation.

Fig 12(B) indicates that when the fractal dimension increases, the energy-absorbing density of the saturated marble becomes greater than that of the dry marble; because water consumes part of the energy that is utilized for destruction, the rock requires additional energy to achieve crushing deformation. In addition, in regard to dry marble, the coefficients pertaining to the quadratic term of the fitted equation are large, and the energy-absorbing density increases at a fast rate, which indicates that the influence that water exerts on the energy-absorbing properties of the rock is gradually decreasing.

## 4. Conclusion

Based on the results of dynamic impact test, which considered the marble that is subjected to dry conditions and the marble that is subjected to saturated conditions, the dynamic parameters and damage fractal characteristics of marble are obtained. The relationship between the two specimens is analyzed, and the following conclusions are obtained:

1. Based on the aforementioned comparative analysis, the Niu Kutou mining area that is located in Qinghai is situated in the highland alpine region, and the marble that characterizes the mining area exhibits different impact dynamics, which distinguishes it from other non-alpine marbles under the effect of multiple freeze–thaws; furthermore, the freeze-thaw effect exerts an apparent weakening effect on the marble.
2. The mechanical properties of saturated marble are the result of the interaction of in-ternal free water weakening and strengthening. When the strain rate is low, the weakening effect of water on rock strength is still dominant, showing that the strength of saturated marble is much lower than that of dry marble. As the strain rate increases, the weakening effect of water gradually decreases. In theory, when the strain rate reaches a threshold, the weakening effect of water on the rock can be completely canceled out, and the saturated rock starts to be stronger than the dry rock.
3. The impact crushing bulkiness of dry marble and saturated marble exhibits similar fractal characteristics, and there is a considerable positive correlation between D and $\dot{\varepsilon }$. When $\dot{\varepsilon }$ < 42.02s^-1^ or $\dot{\varepsilon }$>49.20s^-1^, the degree of fragmentation of dry marble is greater than that of the saturated marble, and the fractal dimension is larger; And when 42.02s^-1^ ≤ $\dot{\varepsilon }$ ≤ 49.20s^-1^, the fractal dimension of the saturated marble is larger than that of the dry marble, which indicates that water exerts different effects on the degree of impact crushing that is exhibited by marble in different strain rate ranges.
4. With respect to both the dry and saturated states, there is a positive correlation between the energy-absorbing density of marble and its fractal dimension, and the energy-absorbing density of the saturated marble is greater than that of the dry marble under a constant fractal dimension. In addition, in regard to both states, when the fractal dimension increases, the energy-absorbing densities of the marble converge, which indicates that when the fractal dimension increases, the influence of water on the energy-absorbing properties of marble decreases.
